# An umbrella meta-analysis of microbial therapy on hepatic steatosis, fibrosis, and liver stiffness in metabolic dysfunction-associated steatotic liver disease

**DOI:** 10.3389/fnut.2025.1686937

**Published:** 2025-11-25

**Authors:** Gvzalnur Kurban, Xiangjun Chen, Xingyi Jin, Hui Xia, Shaokang Wang, Guiju Sun

**Affiliations:** 1 Department of Nutrition and Food Hygiene, Key Laboratory of Environmental Medicine and Engineering of Ministry of Education, School of Public Health, Southeast University, Nanjing, Jiangsu, China; 2 Clinical Medical Research Center for Plateau Gastroenterological Disease of Xizang Autonomous Region, School of Medicine, Xizang Minzu University, Xianyang, China

**Keywords:** MASLD, probiotic, prebiotic, synbiotic, umbrella review

## Abstract

**Background:**

Metabolic dysfunction-associated steatotic liver disease is considered the leading cause of chronic liver disease worldwide. By now, no confirmed medication is accessible for the treatment of MASLD. Previous studies showed the positive effects of microbial therapy, such as probiotics, prebiotics, and synbiotics. The study aims to summarize the results of a meta-analysis of randomized controlled trials and evaluate the impact of microbial therapy (probiotics, prebiotics, and synbiotics) on liver radioactive indicators in patients with MASLD and hopes to bring certain benefits to the adjuvant treatment of MASLD populations.

**Methods:**

A thorough search of PubMed, Scopus, Web of Science, Embase, and Cochrane Library, from inception up to 4 May 2025, was conducted to find meta-analyses on randomized controlled trials reporting the effects of microbial therapy on patients with MASLD. Meta-analyses surveying the impact of microbial therapy on the degree of liver fat infiltration (DFI), hepatic steatosis (HS), hepatic fibrosis (HF), and liver steatosis measurement (LSM) in the MASLD patients were included in our umbrella review. The final effect size (ES) was estimated, and sensitivity and subgroup analyses were performed to explore heterogeneity.

**Results:**

A total of 14 meta-analysis studies were included. The findings demonstrated that microbial therapy could significantly improve hepatic steatosis (measured by ultrasound grading) (HS; OR: 2.612; 95% CI: 1.674, 4.075; *p* < 0.001), hepatic fibrosis (HF; ES: -0.274; 95%CI: −0.427, −0.120; *p* < 0.001), and liver stiffness measurement (LSM; ES: −0.550; 95%CI, −0.716, −0.384; *p* < 0.001) in patients with MASLD.

**Conclusion:**

The present study suggests that microbial therapies seem to be a promising therapeutic approach to the improvement of hepatic steatosis, liver fibrosis, and liver stiffness in patients with MASLD.

**Systematic Review Registration:**

https://www.crd.york.ac.uk/PROSPERO/view/CRD420251043303, identifier PROSPERO (CRD420251043303).

## 1 Introduction

Metabolic dysfunction-associated steatotic liver disease (MASLD) refers to the excessive accumulation of fat in the liver without the influence of significant alcohol consumption (1, 2). MASLD, previously known as non-alcoholic fatty liver disease (NAFLD), is the world’s most common liver disease and a leading cause of liver-related morbidity and mortality. According to the European NAFLD Registry, 98% of its current NAFLD patients meet the revised MASLD criteria (3, 4). MASLD will be used instead of NAFLD throughout this article, and we acknowledge that previous literature has used NAFLD and that they may not be completely the same. Currently, MASLD affects approximately 38% of the global population (5). It is estimated that by 2040, the prevalence of MASLD among adults will rise to over 55% (5). The disease progression of MASLD begins with normal liver function, followed by an imbalance between metabolic inflammatory factors and defensive factors, accompanied by liver steatosis and fatty liver inflammation, leading to liver fibrosis, and ultimately liver stiffness and hepatocellular carcinoma (HCC) (6, 7).

The gut microbiota is the group of microorganisms that inhabits the gastrointestinal tract, which is composed of bacteria, fungi, viruses, and archaea, outnumbering human host cell counts (8). With the significant increase in people’s understanding of the characteristics of the microbiota in metabolic disorders, more and more evidence indicates that changes in the composition and function of the microbiota can affect the metabolic health of human hosts (9–12). A recent review indicates that significant changes in the composition of the microbiome can be observed in the natural history of MASLD and suggests that therapies targeting the gut and microbiota may help regulate the progression of steatohepatitis and fibrosis (13). Li et al. (14) studies show that dealcoholized apple juice sequentially fermented by *Saccharomyces cerevisiae* and *Lactobacillus plantarum* can regulate and restore the intestinal flora, further reduce the production of liver cholesterol and fat accumulation, and promote the production of short-chain fatty acids. Meanwhile, the interaction between polyphenols and the intestinal flora is regarded as a key approach to improving MASLD. A systematic review highlights that polyphenols (which act as prebiotics) can be metabolized by intestinal microorganisms into highly biologically active small molecules and exert a synergistic effect on improving insulin resistance and reducing liver lipid accumulation and inflammation by regulating signaling pathways such as AMPK, PPARs, and NF-κB (15).

These pieces of evidence collectively indicate that interventions targeting the intestinal microbiota ecology can intervene in the disease progression of MASLD through multi-target and multi-pathway approaches. One of the proposed treatments of MASLD is the modulation of the gut microbiome by taking probiotics, prebiotics, and synbiotics (16–18). According to the Food and Agriculture Organization of the United States (FAO), probiotics are defined as a culture of living microorganisms that could provide health benefits for the hosts if consumed in adequate amounts (19, 20). These health benefits include the improvement in barrier function, intestinal stimulation of the immune system, and protection against pathogens (21). In the MASLD mouse model, oxidative stress, inflammation, fibrosis, and carcinogenesis were reduced by probiotics (22). Prebiotics are non-digestible food ingredients that confer a health benefit on the host by selectively stimulating the growth and survival of probiotic bacterial species (23). Examples of prebiotics for humans are the oligosaccharides fructans and galactans, which can stimulate the growth of *Bifidobacteria* and the production of short-chain fatty acids (24). In MASLD disease, prebiotics exert their effects by regulating the composition of the gut microbiota compositions and bile acid metabolism, reducing gut permeability and endotoxemia, and down-regulating the expression of pro-inflammatory cytokines (such as TNF-a, IL-6, and IL-1B) or Toll-like receptors (TLRs) (13). Synbiotics are composed of both probiotics and prebiotics in a form of synergism (25). Synbiotics are can also act on the gut bacterial flora (23).

Although many studies have shown the benefits of microbial therapy for MASLD, the regulatory role of gut microbiota in hepatic fibrosis, liver steatosis, etc., remains controversial. Some meta-analyses have reported that microbial therapy has a significant impact on liver fibrosis and steatosis (26), while others have found no favorable effects (27). Xing et al., in a meta-analysis study, showed that microbial therapies could improve liver steatosis but not liver fibrosis in MASLD patients (28), while Rong et al. (29) demonstrated that microbial therapies had no significant effect on both liver steatosis and liver fibrosis in MASLD patients. Given that the control strategies for hepatic steatosis and liver fibrosis play a significant role in the clinical management of MASLD patients, and due to the inconsistent results of meta-analyses on the regulatory role of the gut microbiome, we aim to review the meta-analyses based on existing evidence-based knowledge. To summarize the effectiveness of the regulatory effect of microbial therapy on radiographic indicators such as hepatic steatosis, liver fibrosis, and stiffness in patients with MASLD disease.

## 2 Materials and methods

### 2.1 Search strategy and study selection

We conducted the present meta-umbrella study evaluating the effects of microbial therapy by administration of prebiotics, probiotics, and synbiotics on the hepatic steatosis, hepatic fibrosis, and liver stiffness of MASLD patients. The reporting of the results was based on the Preferred Reporting Items for Systematic Reviews and Meta-analysis (PRISMA) guideline (30). The review basis and protocol for this umbrella meta-analysis have been registered in the International Prospective Register of Systematic Reviews (PROSPERO: CRD420251043303).^1^

We designed the search strategy based on the population, intervention, comparators, outcomes, and study design(PICOS) format, as shown in Table 1.

**Table 1:** PICOS criteria.

Criterion	Definitions
Population	Patients with metabolic dysfunction-associated steatotic liver disease
Intervention	Probiotics, prebiotics, and synbiotics
Control	Placebo or no treatment
Outcomes	The degree of liver fat infiltration, hepatic steatosis and fibrosis, and liver steatosis.
Study design	Meta-analysis

The scientific international databases, including PubMed, Scopus, Web of Science, Embase, and Cochrane Library, from inception up to 4 May 2025. Given that non-alcoholic fatty liver disease (NAFLD) was renamed MASLD in 2023, we retained NAFLD as the primary keyword for our disease search to ensure consistency with previous literature. The following keywords were used to search the databases: “Nonalcoholic Fatty Liver Disease,” “Probiotics,” “Prebiotics,” “Synbiotics,” “Systematic reviews,” and “Meta-analysis.” To enhance the search quality, information specialists were consulted, and the relevant studies’ reference lists were searched manually. There was no language restriction. No language restriction was imposed. The pattern of search strategy for databases is provided in Supplementary Table 1. We used EndNote 21 for managing the searched articles. The studies searched were uploaded to a systematic review management platform, Rayyan, where secondary duplicate data removal and article screening were conducted.

### 2.2 Inclusion and exclusion criteria

Inclusion criteria included: (1) articles must be meta-analyses or systematic reviews involving meta-analyses and (2) articles must evaluate the effects of probiotics, prebiotics, or synbiotics on patients with MASLD. (3) The article must include the following outcomes: hepatic fibrosis, degree of liver fat infiltration, hepatic steatosis, and/or liver stiffness measurement.

Exclusion criteria included: (1) systematic reviews without meta-analysis and narrative reviews; (2) studies involving participants with multiple diseases other than MASLD; (3) The following studies were excluded: *in vitro*, *in vivo*, and *ex vivo* studies; case reports; observational studies; quasi-experimental studies; and controlled clinical trials.

### 2.3 Quality assessment

The quality of included meta-analyses was assessed by two reviewers (GK and XC) independently using the AMSTAR checklist 2 (31), and any disagreements were resolved by a third researcher (SW). This checklist contains 16 questions with answers “yes,” “no,” or “partial yes.” The final is reported as “high,” “moderate,” “low,” or “critically low” based on the answers of reviewers (32). The quality assessment of the included studies is provided in Supplementary Table 2.

### 2.4 Data extraction

Two reviewers (GK and XJ) independently extracted data from the included studies, and the third researcher (SW) resolved disagreements. The following information were extracted from each study: name of the first author, year, publication of journal, country of study, number of included studies and total sample size, duration of intervention, outcomes of interest, protocol registry number, funding status, methods for assessing the source of heterogeneity and publication bias, model used for analysis, software used for analysis, data bases and date of search, and effect size (ES) and confidential interval (CI) of DFI (the type of effect size, RR), HS (the type of effect size include OR, SMD, MD, and WMD), HF (the type of effect size, SMD), and LSM (the type of effect size include SMD, MD, and WMD). The extracted data were entered into a predesigned Excel sheet.

### 2.5 Data analysis

The Comprehensive Meta-Analysis software version 3 (CMA 3) was used for the graphs and forest plot construction. The effect size (ES) and 95% CI were calculated for each dataset to determine the final effect size. If a study assessed at least two interventions (probiotics, prebiotics, or synbiotics), the data for each intervention were extracted and analyzed as an independent effect size. If an article reported more than one outcome, each outcome was extracted, respectively. The heterogeneity between study associations was estimated through the *I*^2^ statistics and Cochrane’s Q-test. *I*^2^ value greater than 50% or *p*-value less than 0.1 was considered the significance level of between-study heterogeneity. The random-effects model was utilized only when the between-study heterogeneity was significant; otherwise, the fixed-effects model was employed. Subgroup analysis was performed to search for potential sources of interstudy heterogeneity. Sensitivity analysis was conducted to evaluate the result’s stability. Publication bias was evaluated through visual examination of the funnel plot and Egger’s regression test, where the significance level was set to a *p*-value of < 0.1. For any suspected asymmetry in the funnel plot, trim-and-fill analysis was carried out to assess how stable the results were.

## 3 Results

### 3.1 Study selection

**Figure 1:**
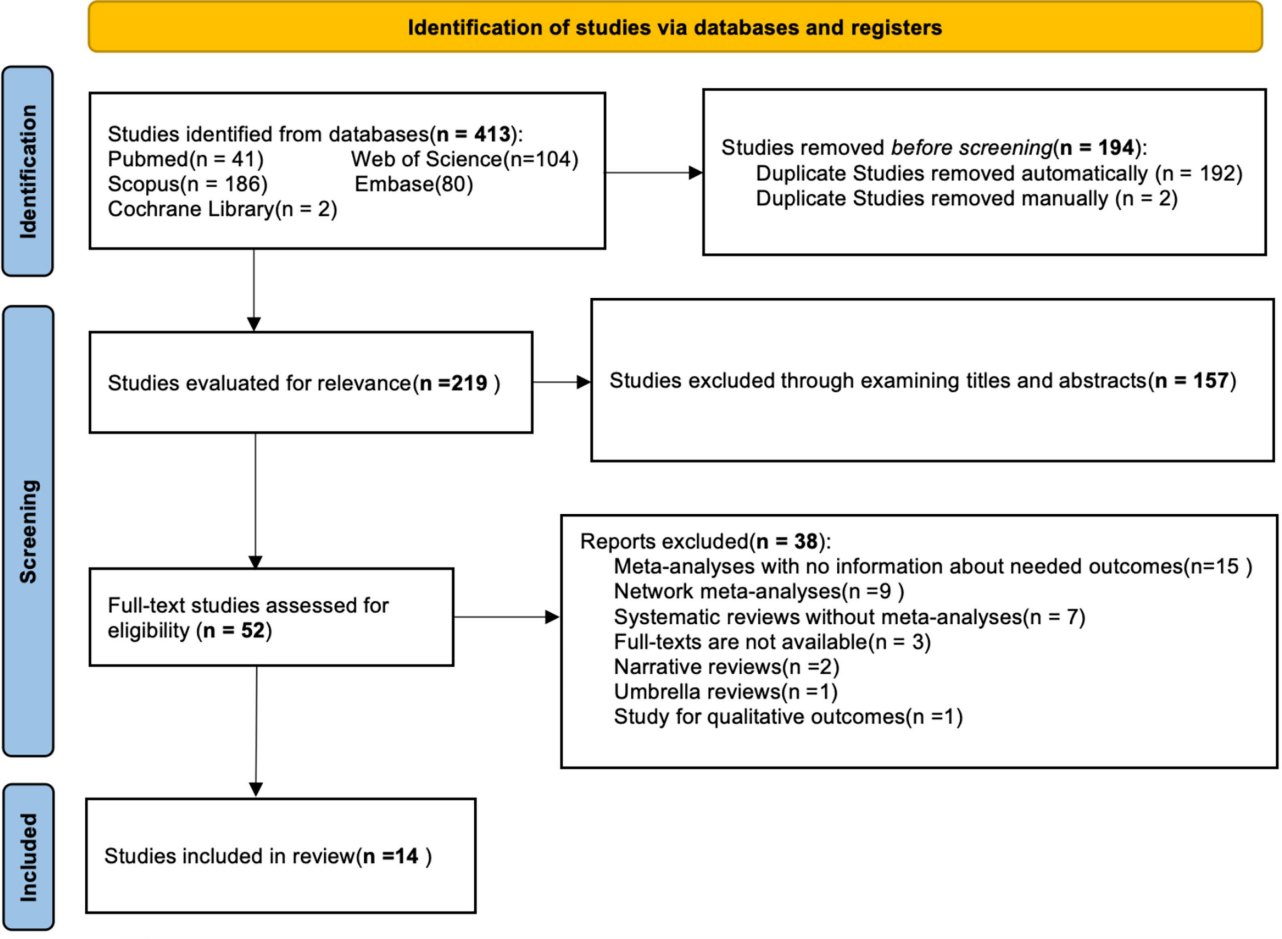
Flow diagram of study selection.

After an initial electronic database search, a total of 413 studies were found, of which 194 were duplicates. After removing 194 duplicates in the first stage, 119 articles remained. Following a thorough review of summaries and titles, 157 articles were excluded. The remaining 52 articles were further screened and classified, resulting in 14 articles being selected for the umbrella meta-analysis. The flow chart shows the study selection process (Figure 1).

### 3.2 Study characteristics

Among the 14 included meta-analyses, 9 were from China, 2 were from the United States, and the 3 remaining were from Iran, Singapore, and Indonesia (n = 1 each). The number of included studies in the meta-analyses and total sample size ranged from 9 to 39 and from 624 to 1,907, respectively. Five studies were registered with PROSPERO. Four studies used probiotics as an intervention, 6 assessed probiotics and synbiotics, 2 assessed synbiotics, and 2 assessed probiotics, prebiotics, and synbiotics. Levels of hepatic steatosis, liver stiffness, hepatic fibrosis, and liver fat infiltration were investigated in 8, 4, 4, and 2 meta-analyses, respectively. Detailed information on all included studies is provided in Supplementary Table 3.

### 3.3 Results of quality assessment

On the basis of the AMSTAR2 checklist, five studies had high quality, five studies had low quality, two had moderate quality, and two had critically low quality. Detailed information on quality assessment is presented in Supplementary Table 2.

### 3.4 Influence of microbial therapy on the level of hepatic steatosis in MASLD patients

#### 3.4.1 Degree of liver fat infiltration

**Figure 2:**
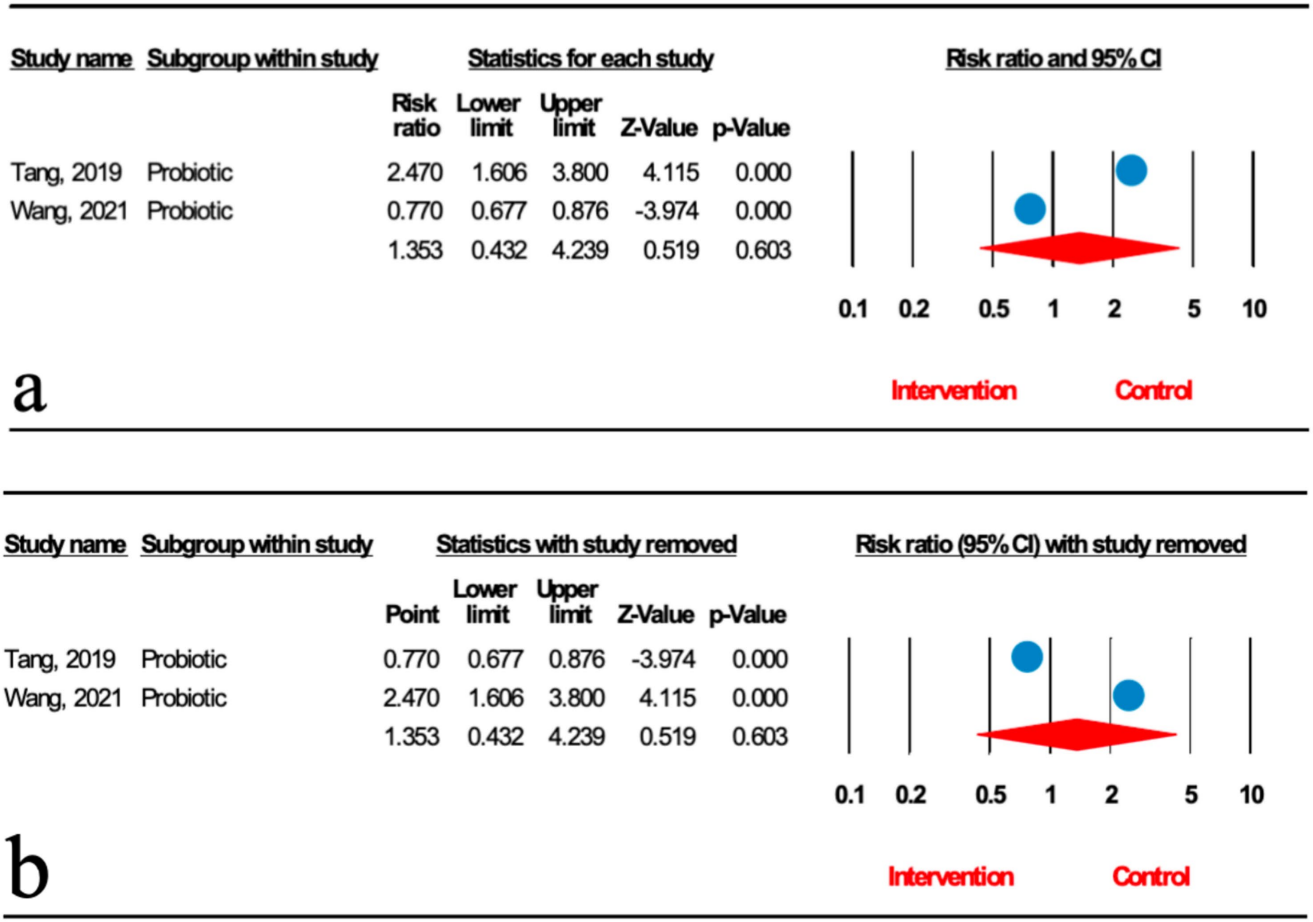
**(a)** Forest plot for the relationship between microbial therapy and the degree of DFI. **(b)** Sensitivity analysis of liver fat infiltration.

Two meta-analyses analyzed the effect of probiotics on the degree of liver fat infiltration (DFI) in MASLD patients. As shown in Figure 2a, the total effect of microbial therapy (probiotic) on serum DFI level was insignificant (relative risk [RR]: 1.353; 95%CI: 0.432, 4.239; *p* < 0.001). Sensitivity analysis results evinced no change after the exclusion of each study (Figure 2b). Significant heterogeneity was noted (*I*^2^ = 96.127%, *p* < 0.001). Subgroup analyses were not conducted due to low number of studies.

#### 3.4.2 Hepatic steatosis measured by ultrasound grading

**Figure 3:**
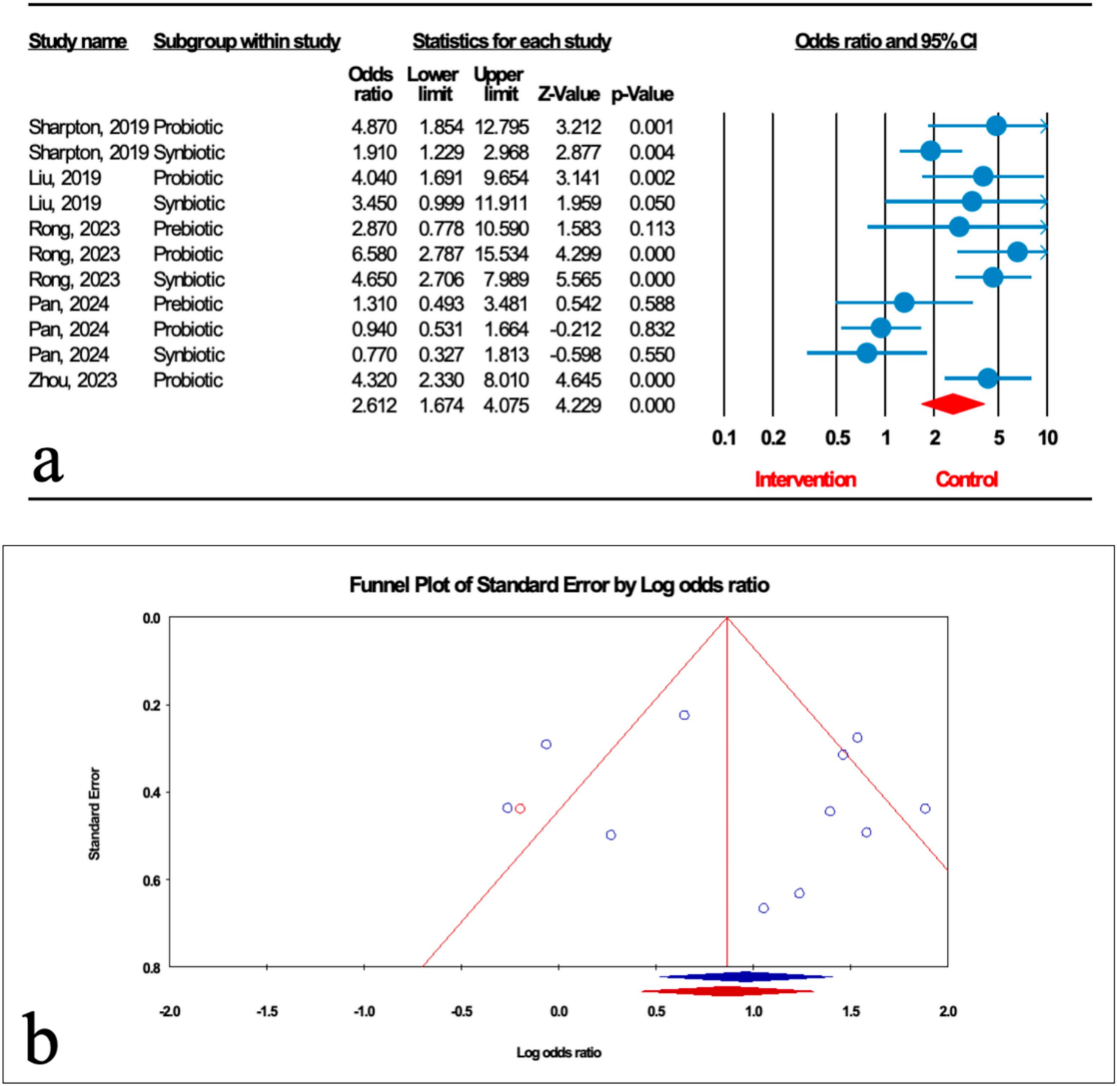
**(a)** Forest plot for the relationship between microbial therapy and the degree of HS. **(b)** The results of publication bias with 1 imputed study (red dot).

As illustrated in Figure 3a, the impact of microbial therapy on hepatic steatosis (HS) as measured by ultrasonographic grading was examined in 11 studies (5 probiotic, 4 synbiotic, and 2 prebiotic studies). Overall, microbial therapy reduced the grade of hepatic steatosis significantly compared with control (odds ratio [OR]: 2.612; 95%CI: 1.674, 4.075; *p* < 0.001). This significant effect was found with probiotic (OR: 2.807; 95%CI: 2.027, 3.886; *p* < 0.001) and synbiotic (OR: 2.348; 95%CI: 1.727, 3.194; *p* < 0.001). However, this relationship was not significant for prebiotics (OR: 1.736; 95%CI: 0.794, 3.796; *p* = 0.167) (Table 2). Sensitivity analysis results evinced no change after the exclusion of each study.

**Table 2:** Results of subgroup analysis with their effect size and 95% confidential interval.

Variables	Subgroups	No. study	Effect size and 95%confidence intervals, *p* value	*I*-squared (%)	*p*-value of heterogeneity
HS^1^					
Total effect		11	2.612;1.674,4.075; <0.001	73.607	<0.001
Intervention type	Probiotic	5	2.807;2.027,3.886;<0.001	81.551	<0.001
	Synbiotic	4	2.348;1.727,3.194;<0.001	78.332	0.003
	Prebiotic	2	1.736;0.794,3.796; 0.167	0.000	0.346
Country	China	3	1.891;1.386,2.581;<0.001	90.504	<0.001
	Other	2	3.624;2.674,4.91;<0.001	80.276	0.024
Previous registered. Protocol	Yes	2	3.624;2.674,4.91;<0.001	80.276	0.024
	No	3	1.891;1.386,2.581;<0.001	90.504	<0.001
Quality of studies	Low	2	4.702;3.365,6.57;<0.001	0.000	0.749
	Moderate	2	1.429;0.997,2.047; 0.052	91.563	0.001
	High	1	2.40;1.50,3.84;<0.001	0.000	1.000
Funded	Yes	4	2.524;2.006,3.177;<0.001	91.025	<0.001
	Not reported	1	3.80;1.958,7.374;<0.001	0.000	1.000
Sample size	<1,000	1	3.80;1.958,7.374;<0.001	0.000	1.000
	≥1,000	4	2.524;2.006,3.177;<0.001	91.025	<0.001
HS^2^					
Total effect		9	-0.546; −1.347,0.256;0.128	74.241	<0.001
Intervention type	Probiotic	3	−0.353; −0.829,0.123; 0.146	0.000	0.724
	Synbiotic	5	−1.102; −1.612, −0.593;<0.001	77.595	0.001
	Prebiotic	1	0.370; −0.280,1.020;0.265	0.000	1.000
Units of reported	MD	2	−30.626;-46.849,-14.403;<0.00	19.963	0.264
	WMD	1	−30.930; −64.520,2.660;0.071	0.000	1.000
	SMD	2	−0.593; −1.038, −0.148;0.009	0.000	0.775
Country	China	2	−0.698; −1.338, −0.059;0.032	92.328	<0.001
	Other	3	−0.548; −1.168,0.071;0.083	55.281	0.107
Previous registered. Protocol	Yes	3	−0.548; −1.168,0.071;0.083	55.281	0.107
	No	2	−0.698; −1.338, −0.059;0.032	92.328	<0.001
Quality of studies	Critically low	1	−17.280; −45.755,11.195;0.234	0.00	1.000
	Low	2	−0.54; −1.16,0.08;0.088	68.208	0.076
	High	2	−0.698; −1.338, −0.059;0.032	92.328	<0.001
Funded	Yes	4	−0.616; −1.061, −0.17;0.007	79.291	0.002
	No	1	−30.930; −64.520,2.66;0.071	0.000	1.000
Sample size	<1,000	2	−0.698; −1.338, −0.059;0.032	92.328	<0.001
	≥1,000	3	−0.548; −1.168,0.071;0.083	55.281	0.107
HF					
Total effect		7	−0.274; −0.427, −0.120;<0.001	10.862	0.346
Intervention type	Probiotic	3	−0.398; −0.639, −0.157;<0.001	35.252	0.213
	Synbiotic	4	−0.189; −0.388,0.010;0.063	0.000	0.589
LSM					
Total effect		9	−0.550; −0.716, −0.384;<0.001	57.179	0.017
Intervention type	Probiotic	4	−0.389; −0.470, −0.307;<0.001	0.000	0.705
	Synbiotic	5	−0.829; −1.049, −0.610;<0.001	0.000	0.452
Units of reported	MD	3	−0.416; −0.525, −0.307;<0.001	65.879	0.053
	SMD	1	−1.17; −2.375,0.035; =0.057	0.000	1.000
	WMD	1	−0.7; −1.00, −0.40;<0.001	0.000	1.000
Country	China	2	−0.377; −0.495, −0.258;<0.001	69.194	0.072
	Other	3	−0.67; −0.868, −0.472;<0.001	0.000	0.660
Previous registered. Protocol	Yes	2	−0.727; −1.019, −0.436;<0.001	0.000	0.458
	No	3	−0.416; −0.525, −0.307;<0.001	65.879	0.053
Quality of studies	Critically low	1	−0.620; −0.890, −0.350;<0.001	0.000	1.000
	Low	1	−0.360; −0.480, −0.240;<0.001	0.000	1.000
	High	3	−0.771; −1.044, −0.498;<0.001	0.000	0.530
Funded	Yes	3	−0.42; −0.531, −0.310;<0.001	71.851	0.029
	No	2	−0.646; −0.910, −0.383;<0.001	0.000	0.383
Sample size	<1,000	2	−0.67; −0.925, −0.414;<0.001	18.791	0.267
	≥1,000	3	−0.413; −0.524, −0.302;<0.001	65.405	0.056

HS^1^: hepatic steatosis measured by ultrasound grading. HS^2^: hepatic steatosis measured by transient elastography. HS, hepatic steatosis; HF, hepatic fibrosis; LSM, liver stiffness measurement; MD, mean difference; SMD, standard mean difference; WMD, weighted mean difference.

There was significant heterogeneity observed when all studies were pooled (*I*^2^ = 73.607%, *p* < 0.001), and the result of subgroup analysis showed that studies with prebiotics as an intervention, studies with low and high quality, studies with not founded, and studies with sample sizes less than 1,000 were accompanied by reduced heterogeneity (*I*^2^ = 0.00%, *p* = 0.346, *I*^2^ = 0.00%, *p* = 0.749, *I*^2^ = 0.00%, *p* = 1.000, *I*^2^ = 0.00%, *p* = 1.000, *I*^2^ = 0.00%, *p* = 1.000, respectively) (Table 2).

The results of Egger’s regression test indicated no significant publication bias (*p* = 0.652); trim-and-fill analysis confirmed robust results with one imputed study (OR: 2.324; 95%CI: 1.886, 2.863) (Figure 3b).

#### 3.4.3 Hepatic steatosis measured by transient elastography

**Figure 4:**
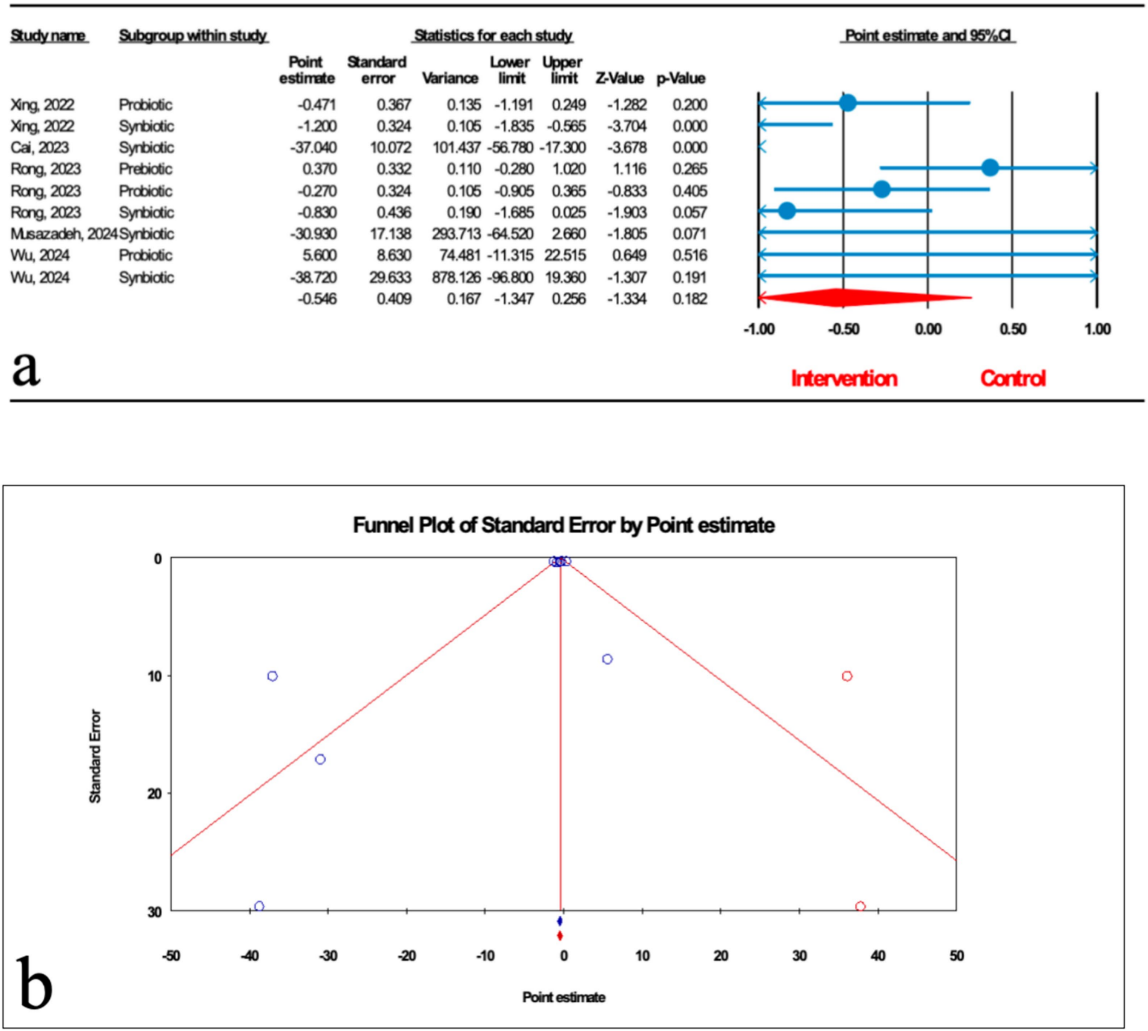
**(a)** Forest plot for relationship between microbial therapy and HS. **(b)** The results of publication bias with two imputed studies (red dots).

As shown in Figure 4a, nine studies (three probiotic, five synbiotic, and one prebiotic studies) assessed hepatic steatosis (HS) severity measured by transient elastography. The results of the umbrella meta-analysis revealed that the total effect of microbial therapy did not significantly improve HS in patients with MASLD (ES: -0.546; 95%CI: −1.347, 0.256; *p* = 0.182). Notably, subgroup analysis demonstrated that synbiotic could significantly reduce HS (ES: -1.102; 95%CI: −1.612, −0.593; *p* < 0.001) (Table 2). The result of the sensitivity analysis confirmed the stability after the exclusion of each study.

There was significant heterogeneity observed when all studies were pooled (*I*^2^ = 74.241%, *p* < 0.001), and the results of subgroup analysis showed that studies with probiotics and prebiotics as intervention, studies that reported their results in MD, WMD and SMD, studies conducted in other counties, studies with previously registered protocol, studies with critically low and low quality, studies with no funding, and studies with sample sizes more than 1,000 were accompanied with decreased heterogeneity (*I*^2^ = 0.00%, *p* = 0.724, *I*^2^ = 0.00%, *p* = 1.000, *I*^2^ = 19.963%, *p* = 0.264, *I*^2^ = 0.00%, *p* = 1.000, *I*^2^ = 0.00%, *p* = 0.775, *I*^2^ = 55.281%, *p* = 0.107, *I*^2^ = 55.281%, *p* = 0.107, *I*^2^ = 0.00%, *p* = 1.000, *I*^2^ = 68.208%, *p* = 0.076, *I*^2^ = 0.00%, *p* = 1.000, *I*^2^ = 55.281%, *p* = 0.107, respectively) (Table 2).

The results of Egger’s regression test indicated no significant publication bias (*p* = 0.124); trim-and-fill analysis confirmed robust results with two imputed studies (ES: -0.453; 95%CI: −0.760, −0.147) (Figure 4b).

### 3.5 Influence of microbial therapy on hepatic fibrosis in MASLD patients

**Figure 5:**
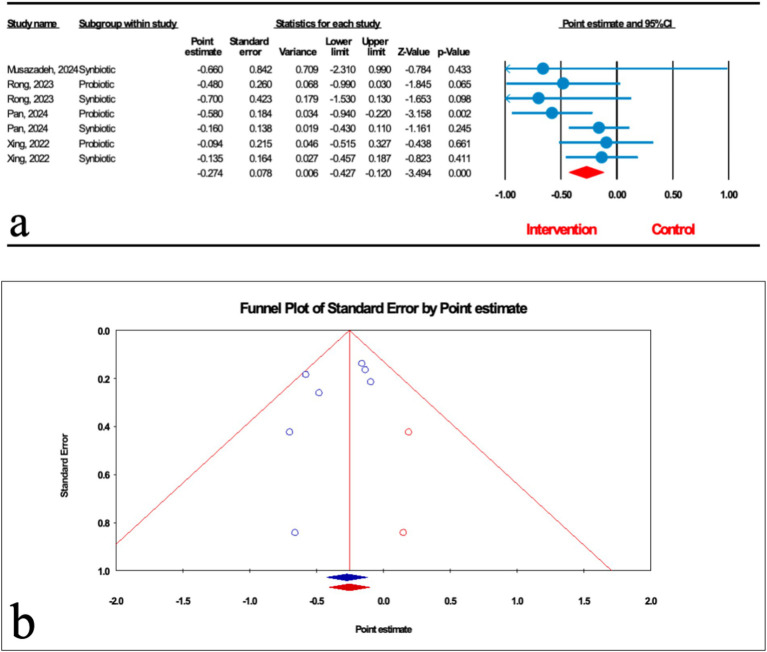
**(a)** Forest plot for the relationship between microbial therapy and the degree of HF. **(b)** The results of publication bias with two imputed studies (red dots).

As demonstrated in Figure 5a, the impact of microbial therapy on hepatic fibrosis (HF) was examined in 7 studies (3 probiotic and 4 synbiotic). Based on the result of our analysis, the total effect of microbial therapy significantly decreased HF in MASLD patients (ES: -0.274; 95%CI: −0.427, −0.120; *p* < 0.001) (Figure 5). According to subgroup analysis, probiotics were shown to have a significant effect on HF (ES: -0.398; 95%CI: −0.639, −0.157; *p* = 0.001), but synbiotic had no significant effect on HF (ES: -0.189; 95%CI: −0.388, 0.01; *p* = 0.063) (Table 2). Sensitivity analysis results confirmed no change after the removal of each study.

The results were homogenous (*I*^2^ = 10.862%, *p* = 0.346). The results of Egger’s regression test indicated no significant publication bias (*p* = 0.244); trim-and-fill analysis confirmed robust results with two imputed studies (ES: -0.254; 95%CI: −0.405, −0.104) (Figure 5b).

### 3.6 Influence of microbial therapy on liver stiffness in MASLD patients

**Figure 6:**
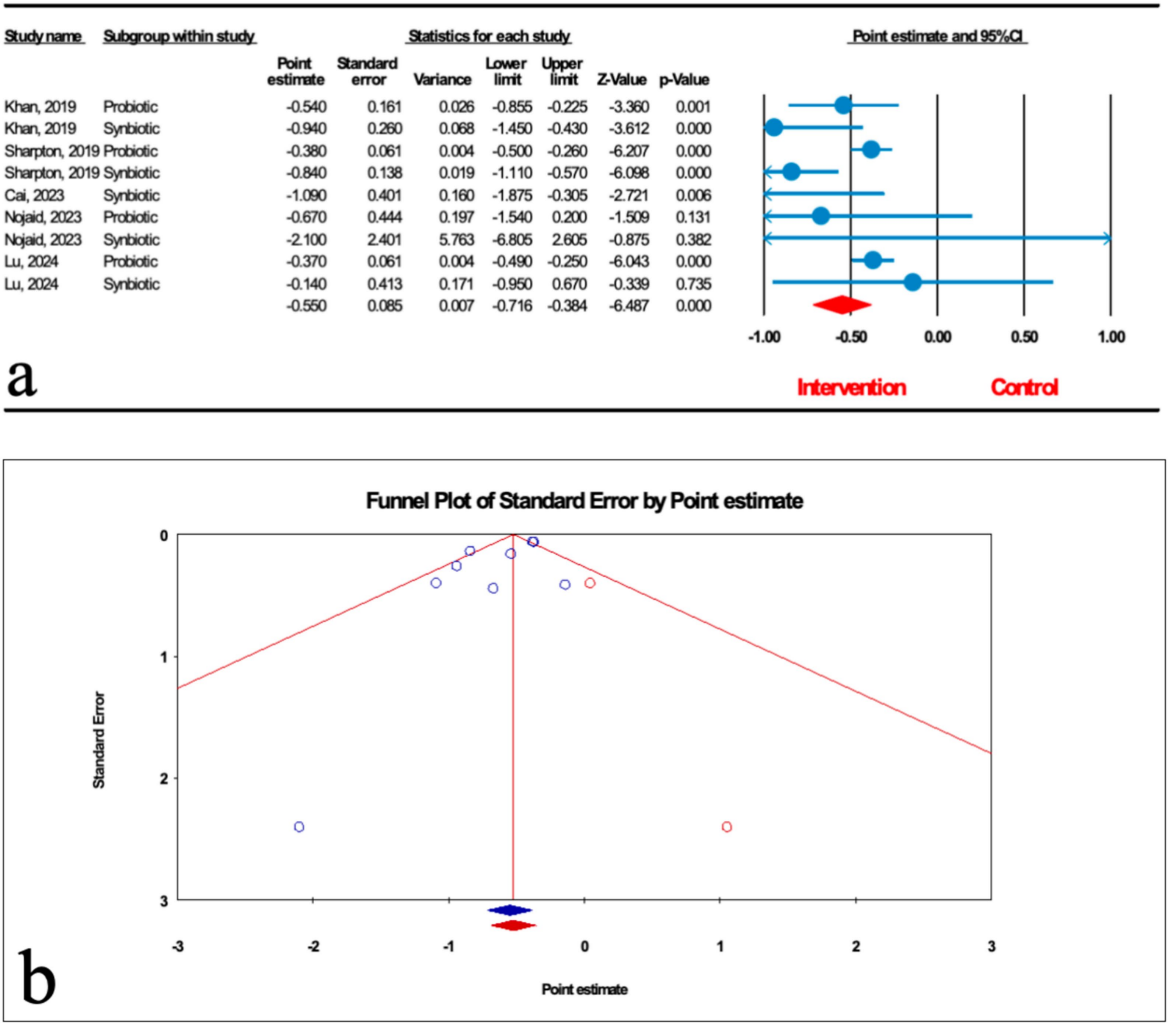
**(a)** Forest plot for the relationship between microbial therapy and the degree of LSM. **(b)** The results of publication bias with two imputed studies (red dots).

Based on the results of nine studies (four probiotic and five synbiotic studies) with effect size, microbial therapy could significantly reduce liver stiffness measurement (LSM) in patients with MASLD (ES: -0.550; 95%CI: −0.716, −0.384; *p* < 0.001) (Figure 6a). Subgroup analysis demonstrated that synbiotics (ES: −0.829; 95%CI: −1.049, −0.610; *p* < 0.001) had the most potent effect on LSM, followed by probiotics (ES: -0.389; 95%CI: −0.470, −0.307; *p* < 0.001) (Table 2). Sensitivity analysis results evinced no change after the exclusion of each study.

There was significant heterogeneity observed when all studies were pooled (*I*^2^ = 57.179, *p* = 0.017), and the result of subgroup analysis showed that studies with probiotics and synbiotics as intervention, studies that reported their results in SMD and WMD, studies conducted in other counties, studies with previously registered protocol, studies with critically low, low and high quality, studies with not founded, and studies with sample sizes less than 1,000 were accompanied with decreased heterogeneity (*I*^2^ = 0.00%, *p* = 0.705, *I*^2^ = 0.00%, *p* = 0.452, *I*^2^ = 0.00%, *p* = 1.000, *I*^2^ = 0.00%, *p* = 1.000, *I*^2^ = 0.00%, *p* = 0.660, *I*^2^ = 0.00%, *p* = 0.458, *I*^2^ = 0.00%, *p* = 1.000, *I*^2^ = 0.00%, *p* = 1.000, *I*^2^ = 0.00%, *p* = 0.530, *I*^2^ = 0.00%, *p* = 0.383, *I*^2^ = 18.791%, *p* = 0.267, respectively) (Table 2).

The results of Egger’s regression test indicated significant publication bias (*p* = 0.076); trim-and-fill analysis confirmed robust results with two imputed studies (OR: 2.324; 95%CI: 1.886, 2.863) (Figure 6b).

## 4 Discussion

MASLD is rapidly becoming one of the most important causes of liver disease. In this umbrella review, we aimed to summarized the effectiveness of microbial therapy (including probiotics, prebiotics, and synbiotics) as a treatment option on patients with MASLD. In conclusion, based on 14 meta-analysis studies, we demonstrated that microbial therapy showed promising effects on hepatic steatosis (HS) measured by ultrasound grading, hepatic fibrosis (HF) and liver stiffness (LSM). We also performed subgroup analysis to assess the effects of probiotics, prebiotics and synbiotics separately. Results indicated that probiotics were most effective in reducing HS measured by ultrasound grading and HF, synbiotics excelled in lowering HS measured by transient elastography and LSM.

Even though our findings showed that microbial therapy may be effective for controlling MASLD, it must be noted that, the results of microbial therapy on HS and LSM were heterogeneous. Differences in intervention type, sample size, study location, units of reported, registered protocol, study quality, fund statement, and sample size may explain this heterogeneity. Furthermore, the results of subgroup implies that microbial therapy in studies with registered protocol can meaningfully improve HS measured by ultrasound grading. Regarding the reduction of LSM, microbial therapy with a high-quality study contributes to a more significant effect. Furthermore, microbial therapy had beneficial effect on reducing HF without any significant heterogeneity.

Human gut microbiota (GM) has numerous contributing bacteria (33). GM dysbiosis, inflammation, and impaired mucosal immune function have a role in MASLD development (34). Probiotics, prebiotics, and synbiotics as an effective and promising therapeutic option for treating MASLD patients, which have been confirmed to have potential role for modulating the gut microbiota (26, 35–37). In this study, we found that probiotics and synbiotics can significantly improve HS and LSM, and probiotics can significantly reduce HF. There are many clinical trials and animal model experiments that are consistent with our results. A placebo-controlled study showed that the supplementation of probiotic formulations Familact (Zisttakhmir, containing seven probiotics) and Fos for 8 weeks could improve hepatic steatosis in MASLD patients (38). In a randomized placebo-controlled trial involving 50 MASLD patients, Mofidi et al. (39) confirmed that synbiotics can significantly reduce hepatic steatosis by transient elastography. In another two randomized trials, using synbiotics for 12 weeks significantly decreased hepatic steatosis (40, 41). In another 24-week randomized clinical trial, synbiotic yogurt consumption reduced hepatic steatosis assessed by abdominal ultrasonography (42). These clinical trial conclusions are consistent with our research findings. Takai et al. (43) showed that supplementation with fructooligosaccharides reduced hepatic steatosis, inflammatory cell infiltration via increased production of short-chain fatty acids in a mouse model of non-alcoholic steatohepatitis. In a rat model of MASLD using high-fructose diet, liver steatosis was reduced by synbiotic formulation (44). Vallianou et al. (45) pointed out in a review that probiotics and synbiotics have been related to a significant decrease in liver stiffness assessed by elastography in patients with MASLD. In addition, a double-blind, placebo-controlled phase II trial involving 104 patients with MASLD showed that a 12-month synbiotic intervention altered the gut microbiota but did not improve liver fibrosis markers (46).

However, other studies reported conflicting results in this regard. In a randomized, double-blind, placebo-controlled trial, 6 months of oral administration of multi-strain probiotics to patients with MASLD did not significantly improve hepatic steatosis or fibrosis (47). In another double-arm, standard treatment-controlled clinical trial treating MASLD patients with probiotic supplementation for 12 months failed to improve fibrosis score (48). Also, the results of a meta-analysis showed that synbiotics supplementation had no significant changes in hepatic fibrosis and hepatic steatosis (27).

A possible explanation for the inconsistent conclusions is that previous studies conducted other interventions (such as lifestyle changes) in addition to microbial therapies. Furthermore, there is no uniform standard for the time of intervention, strain selection, country of patients, intervention duration, number of samples, patient characteristics, and endpoint of the study (49).

This meta-analysis indicated that microbial therapy could be beneficial for individuals with MASLD. The findings are encouraging and suggest that probiotics, prebiotics and synbiotics have a promising future in the treatment of MASLD. While, there are already many promising treatments accessible, such as statins, PPAR agonists, and FXR modulators. Lee et al. (50) summarized that statin use could reduce the chance of getting MASLD and hepatic fibrosis. Wy-14643 is a powerful PPARα agonist that can inhibit steatosis, restore insulin sensitivity, as well as lipid and adiponectin levels, thereby reducing MASLD caused by PPARα dysregulation (51). Clifford et al. (52) demonstrated in mice model that the use of the GSK2324 a FXR agonist reduces lipid uptake and lipid synthesis, thereby reducing hepatic steatosis. However, these pharmacological treatments carry certain side effects for patients. For instance, PPAR agonists increase the risk of bone loss and cardiovascular complications (53), while FXR agonists cause pruritus and elevated blood lipids (52). In comparison, microbiome therapy holds promise as a potentially safer adjunctive treatment.

## 5 Strengths and limitations

This umbrella meta-analysis study systematically summarized the current evidence regarding the effects of microbial therapy on serum levels of DFI, HS, HF, and LSM and also discussed the effects of probiotics, prebiotics, and synbiotics separately through subgroup analysis. In addition, we performed subgroup analyses on the results with high heterogeneity to understand the impact of various factors on the outcomes. However, we also have certain limitations as follows: (1) Some of the articles included in the indicators had a single intervention substance and were few in number. (2) The included studies did not show the side effects of microbial therapy, and its side effects on MASLD are still unclear and need further research. (3) The participants in the articles we included were mainly from Asia, so caution should be exercised when extrapolating the results. (4) In addition, we did not standardize the assessment results of hepatic fibrosis and liver stiffness in the original study. Fiber scanning (transient elastography) may significantly affect the results due to changes in probe type (M and XL), patient position, fasting status, and cutoff values. We strongly recommend that supplementary analysis be conducted in future research. We suggest that well-designed randomized controlled trials are needed to further determine the optimal dosage, treatment duration, and specific microbial strains for microbiome therapy in MASLD.

## 6 Conclusion

In the present study, we found that microbial therapy can significantly improve HS (measured by ultrasound grading), HF, and LSM, but the effect on DFI and HS (measured by transient elastography) was not significant. In the subgroup analysis, probiotics had the most substantial effect on HS, followed by synbiotics. Probiotics had the most significant effect on HF. Synbiotics had the most potent effect on LSM, followed by probiotics. Synbiotics had a significant beneficial effect on HS (measured by ultrasound grading).

However, due to the high heterogeneity of the results, the small number of studies included in each subgroup, and the low quality of most studies, the results of this study should be interpreted with caution.

Furthermore, translating these findings into clinical practice requires addressing core issues such as formulation standardization, dose precision, and rationalization of treatment duration. Future studies should adopt unified efficacy evaluation standards, design multi-center randomized controlled trials, and focus on exploring individualized intervention schemes for different populations to provide high-quality evidence for the development of standardized clinical guidelines.

## Data Availability

The original contributions presented in the study are included in the article/Supplementary material, further inquiries can be directed to the corresponding author.

## Footnotes

1
https://www.crd.york.ac.uk/PROSPERO/view/CRD420251043303
