# Supplementary material for: An umbrella meta-analysis of microbial therapy on hepatic steatosis, fibrosis, and liver stiffness in metabolic dysfunction-associated steatotic liver disease
Source: Front Nutr. 2025 Nov 25;12:1686937. doi: 10.3389/fnut.2025.1686937 (PMC12687379; doi:10.3389/fnut.2025.1686937)
Supplement: Supplementary file 1 [file Supplementary_file_1.zip › Supplement materials/Table 1.docx]

Table 1: Search strategy and keywords of this umbrella review

| PubMed | ((("Non-alcoholic Fatty Liver Disease"[Mesh]) OR (((((((((((((("Non-alcoholic Fatty Liver Disease"[Title/Abstract]) OR ("Non-alcoholic Fatty Liver Disease"[Title/Abstract])) OR ("Non alcoholic Fatty Liver Disease"[Title/Abstract])) OR ("Fatty Liver, Nonalcoholic"[Title/Abstract])) OR ("Fatty Livers, Nonalcoholic"[Title/Abstract])) OR ("Liver, Nonalcoholic Fatty"[Title/Abstract])) OR ("Livers, Nonalcoholic Fatty"[Title/Abstract])) OR ("Nonalcoholic Fatty Liver"[Title/Abstract])) OR ("Nonalcoholic Fatty Livers"[Title/Abstract])) OR ("NAFLD"[Title/Abstract])) OR ("Nonalcoholic Fatty Liver Disease"[Title/Abstract])) OR ("Nonalcoholic Steatohepatitis"[Title/Abstract])) OR ("Steatohepatitides, Nonalcoholic"[Title/Abstract])) OR ("Steatohepatitis, Nonalcoholic"[Title/Abstract]))) AND ((("Prebiotics"[Mesh]) OR ("Probiotics"[Mesh])) OR ("Synbiotics"[Mesh])) AND (meta-analysis[Filter])) AND (("Meta-Analysis"[Publication Type]) OR ("Meta Analysis"[Publication Type])) |
| --- | --- |
| Web of Science | ("Non-alcoholic Fatty Liver Disease" OR "Non alcoholic Fatty Liver Disease" OR "Fatty Liver, Nonalcoholic" OR "Fatty Livers, Nonalcoholic" OR "Liver, Nonalcoholic Fatty" OR "Livers, Nonalcoholic Fatty" OR "Nonalcoholic Fatty Liver" OR "Nonalcoholic Fatty Livers" OR "NAFLD" OR "Nonalcoholic Fatty Liver Disease" OR "Nonalcoholic Steatohepatitis" OR "Steatohepatitides, Nonalcoholic" OR "Steatohepatitis, Nonalcoholic" OR "Metabolic Dysfunction-Associated Steatotic Liver Disease" OR "MASLD") AND ("Synbiotics" OR "Probiotics" and "Prebiotics") AND ("Meta-Analysis" OR "Meta Analysis") |
| Coherence | #1 MeSH descriptOR: [Non-alcoholic Fatty Liver Disease] explode all trees  #2 (Non-alcoholic Fatty Liver Disease):ti,ab,kw OR (Livers, Nonalcoholic Fatty):ti,ab,kw OR (Liver, Nonalcoholic Fatty):ti,ab,kw OR (Fatty Livers, Nonalcoholic):ti,ab,kw OR (Fatty Liver, Nonalcoholic):ti,ab,kw  #3 (Nonalcoholic Fatty Liver Disease):ti,ab,kw OR (Steatohepatitis, Nonalcoholic):ti,ab,kw OR (Non alcoholic Fatty Liver Disease):ti,ab,kw OR (Steatohepatitides, Nonalcoholic):ti,ab,kw OR (Nonalcoholic Fatty Livers):ti,ab,kw  #4 (Nonalcoholic Steatohepatitis):ti,ab,kw OR (Nonalcoholic Fatty Liver):ti,ab,kw OR (Nonalcoholic Steatohepatitides):ti,ab,kw OR (NAFLD):ti,ab,kw  #5 #1 OR #2 OR #3 OR #4 |
| Scopus | (TITLE-ABS-KEY( "Non-alcoholic Fatty Liver Disease" OR "Nonalcoholic Fatty Liver Disease" OR "Fatty Liver, Nonalcoholic" OR "Fatty Livers, Nonalcoholic" OR "Liver, Nonalcoholic Fatty" OR "Livers, Nonalcoholic Fatty" OR "Nonalcoholic Fatty Liver" OR "Nonalcoholic Fatty Livers" OR "NAFLD" OR "Nonalcoholic Fatty Liver Disease" OR "Nonalcoholic Steatohepatitis" OR "Steatohepatitides, Nonalcoholic" OR "Steatohepatitis, Nonalcoholic" OR "Metabolic Dysfunction-Associated Steatotic Liver Disease" OR "MASLD" ) AND TITLE-ABS-KEY ( "Synbiotics" OR "Probiotics" OR "Prebiotics" ) AND TITLE-ABS-KEY ( "meta-analysis" OR "Systematic Review" ) ) |
| Embase | #1 'nonalcoholic fatty liver'/exp  #2 'probiotic agent'/exp  #3 'prebiotic agent'/exp  #4 'prebiotic agent':ab,ti OR prebiotic:ab,ti OR prebiotics:ab,ti  #5 'probiotic agent':ab,ti OR probiotic:ab,ti OR probiotics:ab,ti  #6 'synbiotic agent'/exp  #7 'synbiotic agent':ab,ti OR synbiotic:ab,ti OR synbiotics:ab,ti  #8 #2 OR #3  #9 #4 OR #5  #10 #6 OR #7  #11 'meta analysis'/exp  #12 'analysis, meta':ab,ti OR metaanalysis:ab,ti OR 'meta analysis':ab,ti  #13 #11 OR #12  #14 #8 OR #9 OR #10  #15 nafld:ab,ti OR 'non alcoholic fatty liver disease':ab,ti OR 'non alcoholic hepato-steatosis':ab,ti OR 'non alcoholic hepatosteatosis':ab,ti OR 'non alcoholic liver steatosis':ab,ti OR 'non alcoholic steatotic hepatopathy':ab,ti OR 'non-alcoholic fatty liver':ab,ti OR 'non-alcoholic fatty liver disease':ab,ti OR 'non-alcoholic fld':ab,ti OR 'non-alcoholic hepatic steatosis':ab,ti OR 'nonalcoholic fatty liver disease':ab,ti OR 'nonalcoholic fld':ab,ti OR 'nonalcoholic hepatic steatosis':ab,ti OR 'nonalcoholic hepatosteatosis':ab,ti OR 'nonalcoholic liver steatosis':ab,ti OR 'nonalcoholic fatty liver':ab,ti  #16 #1 OR #15  #17 #13 AND #14 AND #16 |
