# Supplementary material for: An umbrella meta-analysis of microbial therapy on hepatic steatosis, fibrosis, and liver stiffness in metabolic dysfunction-associated steatotic liver disease
Source: Front Nutr. 2025 Nov 25;12:1686937. doi: 10.3389/fnut.2025.1686937 (PMC12687379; doi:10.3389/fnut.2025.1686937)
Supplement: Supplementary file 1 [file Supplementary_file_1.zip › Supplement materials/Table 3.docx]

Table 3. Information of included studies.

| **Auther/Year** | **Name of the Journal** | **Country** | **Number of included studies/total sample size** | **Intervention*/duration of treatment** | **Outcomes** | **Protocol registry** | **Funding status** | **Source of heterogeneity assessment methods** | **Risk of bias assessment tool** | **Publication bias assessment method** | **Model used for analysis** | **Software used for analysis** | **Data bases/ date of search** | **Quality assessment score** |
| --- | --- | --- | --- | --- | --- | --- | --- | --- | --- | --- | --- | --- | --- | --- |
| Khan et al (2019)(1) | European Journal of Gastroenterology & Hepatology | USA | 12 / 624 | Probiotics and synbiotics;not reportred | LSM | Not reported | No funding | Sensitivity analysis, subgroup analyses | Cochrane | No assessment | Random-effects model | RevMan | PubMed, Medline, and Google Scholar; up to 10 June 2018 | Critically low |
| Liu et al (2019)(2) | Digestive Diseases and Sciences | China | 15 / 782 | Probiotics and synbiotics;no reportred | HS | Not reported | Not reported | Sensitivity analysis, subgroup analyses | Cochrane | No assessment | Random-effects model | RevMan | PubMed, Cochrane, and Embase; up to April 2018 | Moderate |
| Sharpton et al (2019)(3) | The American Journal of Clinical Nutrition | USA | 21 / 1252 | Probiotics and synbiotics;no reportred | LSM, HS | (PROSPERO) CRD42018091455 | Founded | Sensitivity analyses, meta-regression, subgroup analyses | Cochrane | Begg's rank correlation test and Egger's regression test | Random-effects model | STATA | PubMed, MEDLINE, EMBASE, and the Cochrane Library;from January 1, 2005 to December 1, 2018 | High |
| Tang et al (2019)(4) | Therapeutic Advances in Gastroenterology | China | 22 / 1356 | Probiotics; 4 to 24 weeks | DFI | Not reported | NFounded | Sensitivity analysis, subgroup analyses | Cochrane | Egger’s test | Fixed effect model for nonheterogenic and random effects model for heterogenic studies | STATA | PubMed, Embase, the Cochrane Library, and the Web of Science; the Chinese databases searched included the China National Knowledge Infrastructure (CNKI), Wan Fang Data, and VIP Database;up to 8 April 2019 | High |
| Wang et al (2021)(5) | Acta Nutrimenta Sinica | China | 9 / 694 | Probiotics;4 to 48 weeks | DFI | Not reported | Founded | Sensitivity analysis, subgroup analyses | Cochrane | No assessment | Fixed effect model for nonheterogenic and random effects model for heterogenic studies | RevMan | PubMed, Embase, Cochrane library and Web of Science; up to 10 March 2021 | Low |
| Xing et al (2022)(6) | Frontiers in Nutrition | China | 11 / 741 | Probiotics and synbiotics;2 to 14 months | HS, HF | Not reported | Founded | Sensitivity analysis, subgroup analyses | Cochrane | Egger’s test , Begg’s test, and funnel plots | Fixed effect model for nonheterogenic and random effects model for heterogenic studies | STATA | Pubmed, PMC, ISI Web of Science, Embase, Cochrane Library, and Chinese National Knowledge Infrastructure (CNKI); between January 2020 and Jul 2022 | High |
| Cai et al (2023)(7) | Therapeutic Advances in Gastroenterology | China | 10 / 624 | Synbiotics;8 weeks to 14 months | LSM, CAP | Not reported | Founded | Subgroup analyses, meta-regression | Cochrane | Funnel plots and Egger’s test | Fixed effect model for nonheterogenic and random effects model for heterogenic studies | RevMan | PubMed, Embase, Cochrane Library, and Web of Science; up to 1 September 2022 | High |
| Nojaid et al (2023)(8) | Human Nutrition and Metabolism | Indonesia | 20 / 1204 | Probiotics, and synbiotics;2 to 14 months | LSM | (PROSPERO) CRD42023392048 | No funding | Subgroup analysis | Cochrane | Funnel plot | Random-effects model | RevMan | PubMed, CENTRAL, Taylor & Francis, EBSCO, ProQuest, and ScienceDirect; up to December 20, 2022 | High |
| Rong et al (2023)(9) | Journal of Gastroenterology and Hepatology | Singapore | 39 / 2563 | Probiotics, prebiotics, and synbiotics;2 to 14 months | HS, HF | (PROSPERO) CRD42022354562 | Founded | Sensitivity analysis, subgroup analyses | Cochrane | No assessment | Random-effects model | RevMan | Medline, Embase, Cochrane Library, EBSCO, and Scopus； up to August 19, 2022 | Low |
| Zhou et al (2023)(10) | Medicine (Baltimore) | China | 21 / 1037 | Probiotics;8 to56 weeks | HS | Not reported | Founded | Sensitivity analysis, subgroup analyses | Cochrane | No assessment | Fixed effect model for nonheterogenic and random effects model for heterogenic studies | RevMan | EMbase, PubMed, Web of Science, and Cochrane; up to April 6, 2022. | Low |
| Lu et al (2024)(11) | China Pharmacy | China | 24 / 1391 | Probiotics and synbiotics;not reportred | LSM | Not reported | Founded | Sensitivity analysis, subgroup analyses | Cochrane | Egger’s test | Fixed effect model for nonheterogenic and random effects model for heterogenic studies | RevMan | PubMed, Embase, Web of Science, Cochrane, Chinese National Knowledge Infrastructure (CNKI), Wan Fang Data, VIP Database，Google Scholar and Baidu Scholar; up to 10 October 2023 | Low |
| Musazadeh et al (2024)(12) | Pharmacological Research | Iran | 18 / 1188 | Synbiotics;8 to 56 weeks | HS, HF | (PROSPERO) CRD42024574719 | No funding | Sensitivity analysis, subgroup analyses | Cochrane | Funnel plot, Begg's adjusted rank correlation, Egger's regression asymmetry tests and trim and fill | Random-effects model | STATA | Embase, PubMed, Web of Science, Cochrane Library, and Scopus; up to June 2024 | Low |
| Pan et al (2024)(13) | BMC Gastroenterology | China | 34 / 1907 | Probiotics, prebiotics, and synbiotics;4 to 56 weeks | HS, HF | Not reported | Founded | Meta-regression, subgroup analyses | Cochrane | Funnel plots, The Begg’s and Egger’s test | Fixed effect model for nonheterogenic and random effects model for heterogenic studies | STATA and RevMan | Embase, PubMed, Cochrane Library, and Web of Science; up to March 2024 | Moderate |
| Wu et al (2024)(14) | Clinics and Research in Hepatology and Gastroenterology | China | 21 / 1489 | Probiotics;8 to 56 weeks | HS | (PROSPERO) CRD42023445554 | Founded | Sensitivity analysis, subgroup analyses | Cochrane | Egger's test | Fixed effect model for nonheterogenic and random effects model for heterogenic studies | RevMan | PubMed/Medline, Web of Science, Embase, and Cochrane Library; from January 1, 2012, to July 15, 2023 | Critically low |

* Intervention and duration of treatment for the outcomes of interest.

Abbreviations: HF, hepatic fibrosis; LSM, liver stiffness measurement; HS, hepatic steatosis; DFI, degree of liver fat infiltration; CNKI, China National Knowledge Infrastructure; USA, United States of America.

1. Khan MY, Mihali AB, Rawala MS, Aslam A, Siddiqui WJ. The promising role of probiotic and synbiotic therapy in aminotransferase levels and inflammatory markers in patients with nonalcoholic fatty liver disease - a systematic review and meta-analysis. Eur J Gastroenterol Hepatol. 2019;31(6):703-15.

2. Liu L, Li P, Liu Y, Zhang Y. Efficacy of Probiotics and Synbiotics in Patients with Nonalcoholic Fatty Liver Disease: A Meta-Analysis. Dig Dis Sci. 2019;64(12):3402-12.

3. Sharpton SR, Maraj B, Harding-Theobald E, Vittinghoff E, Terrault NA. Gut microbiome-targeted therapies in nonalcoholic fatty liver disease: a systematic review, meta-analysis, and meta-regression. Am J Clin Nutr. 2019;110(1):139-49.

4. Tang Y, Huang J, Zhang WY, Qin S, Yang YX, Ren H, et al. Effects of probiotics on nonalcoholic fatty liver disease: a systematic review and meta-analysis. Therapeutic Advances in Gastroenterology. 2019;12.

5. Wang Y, Bian X, Yuan M, Wan M, Gao W, Guo C. EFFECTS OF PROBIOTICS ON NON-ALCOHOLIC FATTY LIVER DISEASES: A META-ANALYSIS. Acta Nutrimenta Sinica. 2021;43(5):442-9,55.

6. Xing W, Gao W, Lv X, Zhao Z, Mao G, Dong X, et al. The effects of supplementation of probiotics, prebiotics, or synbiotics on patients with non-alcoholic fatty liver disease: A meta-analysis of randomized controlled trials. Frontiers in Nutrition. 2022;9.

7. Cai J, Dong J, Chen D, Ye H. The effect of synbiotics in patients with NAFLD: a systematic review and meta-analysis. Therapeutic Advances in Gastroenterology. 2023;16.

8. Nojaid A, William J, Tanjaya K, Taslim NA, Nurkolis F. Using synbiotics supplementation to treat hepatic steatosis: A comprehensive meta-analysis of randomized controlled trials. Human Nutrition and Metabolism. 2023;32.

9. Rong L, Ch'ng D, Jia P, Tsoi KKF, Wong SH, Sung JJY. Use of probiotics, prebiotics, and synbiotics in non-alcoholic fatty liver disease: A systematic review and meta-analysis. J Gastroenterol Hepatol. 2023;38(10):1682-94.

10. Zhou X, Wang J, Zhou S, Liao J, Ye Z, Mao L. Efficacy of probiotics on nonalcoholic fatty liver disease: A meta-analysis. Medicine (Baltimore). 2023;102(4):e32734.

11. Lu F, Xing G, Hu S. Efficacy and safety of probiotics in the treatment of nonalcoholic fatty liver disease：a meta-analysis. China Pharmacy. 2024;35(13):1643-50.

12. Musazadeh V, Assadian K, Rajabi F, Faghfouri AH, Soleymani Y, Kavyani Z, et al. The effect of synbiotics on liver enzymes, obesity indices, blood pressure, lipid profile, and inflammation in patients with non-alcoholic fatty liver: A systematic review and meta-analysis of randomized controlled trials. Pharmacol Res. 2024;208:107398.

13. Pan Y, Yang Y, Wu J, Zhou H, Yang C. Efficacy of probiotics, prebiotics, and synbiotics on liver enzymes, lipid profiles, and inflammation in patients with non-alcoholic fatty liver disease: a systematic review and meta-analysis of randomized controlled trials. BMC Gastroenterol. 2024;24(1):283.

14. Wu J, Chen X, Qian J, Li G. Clinical improvement effect of regulating gut microbiota on metabolic dysfunction-associated steatotic liver disease: Systematic review and meta-analysis of randomized controlled trials. Clin Res Hepatol Gastroenterol. 2024;48(7):102397.
